# Significance of Estrogen/Progesterone Receptor Expression in Metaplastic Breast Carcinoma

**DOI:** 10.1155/2024/2540356

**Published:** 2024-04-03

**Authors:** Atif Ali Hashmi, Bakhtawar Allauddin Mallick, Khushbakht Rashid, Umair Arshad Malik, Shamail Zia, Fazail Zia, Muhammad Irfan

**Affiliations:** ^1^FRCPath, Department of Histopathology, Liaquat National Hospital and Medical College, Karachi 74800, Pakistan; ^2^Zainab Panjwani Memorial Hospital, Karachi 74800, Pakistan; ^3^Emergency Medicine, Al-Rayaz Hospital, Karachi 75850, Pakistan; ^4^Prime Cardiology of Nevada, Las Vegas 89128, USA; ^5^Department of Nephrology, Sindh Institute of Urology and Transplantation, Karachi 74200, Pakistan; ^6^Department of Internal Medicine, Aga Khan University, Karachi 74800, Pakistan; ^7^Department of Pathology, Jinnah Sindh Medical University, Karachi 75510, Pakistan; ^8^Department of Biostatistics, Liaquat National Hospital and Medical College, Karachi 74800, Pakistan

## Abstract

**Introduction:**

Metaplastic breast carcinoma (MBC) is a rare subgroup of breast neoplasms associated with adverse outcomes because of its aggressive nature. Typically, MBCs show triple-negative hormone receptor (HR) status. Determining the HR status of breast cancer is an integral part because it is an important prognostic factor and helps in the treatment course of the disease. This study aimed to determine the HR status of MBC, its significance, and its association with various clinicopathological parameters.

**Methods:**

This was a retrospective study conducted at the Department of Histopathology, Liaquat National Hospital. A total of 140 biopsy-proven cases of MBC were enrolled in the study. Clinical and pathological data were retrieved from the institutes' archives. Immunohistochemical studies were conducted to determine the estrogen receptor (ER) and progesterone receptor (PR) status.

**Results:**

The mean age of MBC in our population was found to be 52.18 ± 12.19 years. The HR positivity rate in our population was found to be 32.9%. A significant association was found between HR status and tumor laterality, tumor size, tumor grade, tumor stage, and recurrence. ER/PR-negative MBCs were most probably associated with higher grade and higher tumor stage and were larger in size (6.62 ± 3.43 cm) than ER/PR-positive MBCs (4.20 ± 1.88 cm). Moreover, ER/PR-positive MBCs showed a higher recurrence rate than ER/PR-negative MBCs (43.5% vs. 25.5%, respectively). No statistically significant relationship was found between HR status and patient age, histological subtype, or survival rate.

**Conclusion:**

MBC is a rare breast neoplasm. MBC was found to be triple negative in most cases, but a significant percentage were HR (ER/PR) positive. Moreover, we found an association between HR status and various clinicopathological features, indicating that HR status is a significant predictor of MBC prognosis.

## 1. Introduction

Metaplastic breast carcinoma (MBC) is a rare type of breast cancer with a highly aggressive nature and is associated with high mortality and morbidity [1]. It constitutes 1% of all invasive breast cancers [2]. MBCs are heterogeneous neoplasms that display a unique morphological presentation with epithelial and mesenchymal components. It is characterized by a partial or complete transformation of glandular epithelium to nonglandular epithelium, such as squamous epithelium or mesenchymal component (such as chondroid, spindle, osseous differentiation, or others) [3].

Clinically, these tumors present as large tumors, with sizes ranging from 1.2 to 10 cm and are often palpable breast masses with ill-defined borders on imaging [1]. These tumors are usually considered to present at a higher grade and stage and are hormone receptor (HR)-negative with a higher propensity for distant metastasis and paucity of regional lymph node involvement compared with invasive breast cancers [4]. Furthermore, these tumors disseminate via a hematogenous route rather than lymphatically [5].

Immunohistochemical study to determine the HR status of the tumor is an essential part of the diagnosis of breast cancer because it is a significant prognostic indicator and key factor in determining the line of treatment [6]. It has been well established that in most cases (>90%) these tumors are triple-negative, that is, they are negative for estrogen receptor (ER) and progesterone receptor (PR) and do not overexpress epidermal growth factor receptor 2 (HER2/neu), with a high tendency for local relapse and distant metastasis [5, 7]. Because of their triple-negative status, these tumors cannot be effectively treated with targeted antiestrogens or therapies targeting the HER2/neu receptor because of their adverse outcomes [8]. The guidelines of the National Comprehensive Cancer Network (NCCN) recognize metaplastic neoplasms as an independent, poor prognostic indicator because patients with MBC show worse outcomes than patients with nontriple negative (NTN), nonmetaplastic breast cancers [9].

Although a rare variant of breast cancer, these cancers pose a significant challenge in their treatment because of their diverse morphological presentation and aggressive nature. Hence, it is essential to better understand the disease and its molecular alterations and to determine the prognostic factors that may help in designing a more targeted treatment modality. Very few studies have been conducted in our population on the significance of HR status in MBC and its association with various clinicopathological parameters. This study provided an overview of the clinical and pathological characteristics and determined the significance of HR status (ER/PR) in MBC.

## 2. Methods

We conducted a retrospective, cross-sectional study at the Department of Histopathology, Liaquat National Hospital, Karachi, Pakistan. A total of 140 cases of MBC reported at the institute over a period of 12 years were enrolled in the study. Clinicopathological data on MBC reported between August 2011 and July 2023 were retrieved from the institute archive. All biopsy-proven MBC cases were included in this study. After clinical examination and workup, including computed tomography (CT) scan and incisional biopsy, all patients underwent surgical resection of the primary tumor at our hospital. Cases missing clinical and surgical records were excluded from the study. Moreover, patients who underwent neoadjuvant chemotherapy or radiotherapy before surgical resection were excluded from the study. The specimens obtained during surgery were sent to the laboratory, and after gross examination, the obtained samples were prepared for histological and immunohistochemical (IHC) staining to evaluate the ER/PR status.

### 2.1. Sample Selection

Samples were obtained by one of the following four methods: trucut biopsy, modified radical mastectomy, breast conversion surgery, or simple mastectomy. After receiving the resected samples at the histopathology laboratory, a gross examination of the samples was performed to note the size, texture, color, and appearance of the tumor.

### 2.2. Histological Sample Preparation

For histological examination, paraffin-embedded tissue blocks were prepared by overnight fixation of the specimen in 10% neutralized formalin, which were then washed and dehydrated by treatment with increasing concentration of alcohol to remove water from the specimens. The tissue samples were then treated with xylene for 3 hr to remove alcohol and immersed in paraffin wax at 56°C. The formalin-fixed-paraffin embedded blocks were then sliced into 3–4 *μ*m sections. Sliced sections were transferred onto L-lysin-treated slides, sequentially treated with xylene, alcohol, and water, and stained with hematoxylin and eosin [10]. These slides were examined by a senior histopathologist at the institute. Histological features, such as tumor differentiation, tumor grade, tumor stage, and tumor size were studied.

### 2.3. Immunohistochemical Sample Preparation

**Figure 1:**
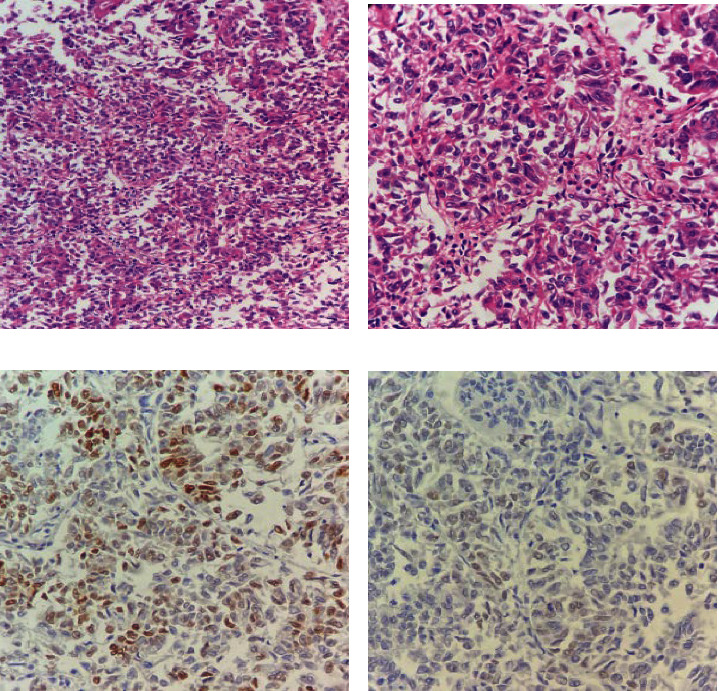
Metaplastic carcinoma with squamous differentiation. (a) Hematoxylin and eosin (H & E)-stained sections at 200x magnification showing solid tumor growth. (b) H & E-stained section at 400x magnification showing squamoid differentiation. (c) Estrogen receptor (ER) staining at 400x magnification showing intermediate nuclear expression. (d) Progesterone receptor (PR) staining at 400x magnification showing weak nuclear expression.

**Figure 2:**
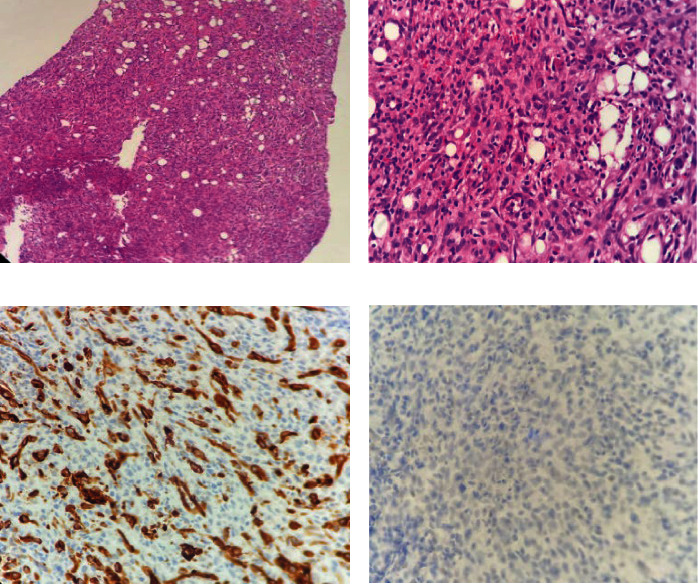
Metaplastic carcinoma with spindle cell differentiation. (a) Hematoxylin and eosin (H & E)-stained sections at 200x magnification showing sheets of spindle cells. (b) H & E-stained section at 400x magnification showing atypical spindled cells. (c) Pan-cytokeratin (CKAE1/AE3) staining at 400x magnification showing positive staining in spindle cells. (d) Estrogen receptor (ER) staining at 400x magnification showing lack of nuclear expression.

For IHC studies of ER/PR and HER2/neu expression, the DAKO envision kit was used. Two DAKO antibodies, ready to use (RTU) Monoclonal Rabbit Anti-human Estrogen receptor alpha (clone EP1) and RTU Monoclonal Mouse Anti-human Progesterone receptor (clone PgR 636) were used. IHC was performed according to the manufacturer's instructions. Paraffin-embedded tissue blocks were deparaffinized, sectioned, and stained automatedly. Normal breast tissue was considered a positive control for ER/PR, and staining of more than 1% for ER/PR was considered a positive expression (Figures 1 and 2). Complete membranous expression of HER2/neu in more than 10% invasive cancer cells was considered positive. Cases with equivocal HER2/neu were confirmed using fluorescence in situ hybridization (FISH).

### 2.4. Statistical Analysis

Data analysis was performed using the Statistical Package for the Social Sciences. (SPSS, Version 26.0; IBM Inc., Armonk, NY, USA). The mean and standard deviation for patient age, tumor size, and follow-up duration were calculated, and a *t*-test was used to determine statistical significance. Moreover, the frequencies and percentages of all other clinicopathological variables were calculated. *Chi*-square and Fisher's exact tests were applied to determine the association between ER/PR expression and clinicopathological features. A *p*-value of 0.05 was considered significant.

## 3. Results

### 3.1. Demographic and Clinicopathological Parameters of Population under Study

A total of 140 cases of MBC were included in the study.  Table 1 illustrates the clinicopathological parameters of the patients included in the study. The mean age of the patients was found to be 52.18 ± 12.19 years, and the disease was more prevalent among the older population of >50 years. The mean follow-up duration was found to be 4.14 ± 1.88 years. MBC was found to be more prevalent on the right side in our population, accounting for 55.7% of cases, whereas 44.3% of cases were those of MBC affecting the left breast. The mean tumor size was found to be 5.99 ± 3.44 cm. Samples were obtained by trucut biopsy (48 cases), modified radical mastectomy (40 cases), simple mastectomy (30 cases), or breast conversion surgery (22 cases). Most cases (50%) were found to be at tumor stage 3 (T3), with 37% of cases found to be at the T2 stage. The majority of the cases were found to be grade 3 tumors (75.7%), whereas the remaining 24.3% of cases were found to be grade 2 tumors. Squamous differentiation in tumors was observed in 68.6% of cases, whereas spindle cell differentiation was present in only 11.4% of cases and chondroid differentiation was present in 5.7% of cases. The most common nodal stage of tumor in our population was N0, noted in 56.5% of cases, followed by N1, present in 19.6% of cases. Recurrence was noted in 31.4% of the cases, with a survival rate of 71.4%. ER/PR was positive in 32.9% of the cases. HER2/neu positive expression was noted in 22.9% cases.

### 3.2. Association of Clinicopathological Parameters with Biomarker Studies

Table 2 illustrates that the association of various clinicopathological features with ER/PR expression. A statistically significant correlation was established between tumor laterality, tumor size, tumor grade, tumor stage, and recurrence with ER/PR expression. We concluded that ER/PR-positive MBCs more commonly affected the left side of the breast, whereas ER/PR-negative MBCs more commonly affected the right side (61.7%). Similarly, ER/PR-positive MBCs were most likely to be smaller in size (4.20 ± 1.88 cm) than ER/PR-negative MBCs (6.62 ± 3.43 cm). Similarly, ER/PR-positive MBCs were most likely of lower grade than ER/PR-negative (grade 2-43.5% vs. 14.9%, Grade 3-56.5% vs. 85.1%, respectively). Moreover, ER/PR-negative MBCs presented at a higher stage than ER/PR-positive MBCs (T1–11.8% vs. 16.7%, T2-26.5% vs. 66.7%, T3- 61.8% vs. 16.7%, respectively). We also concluded that ER/PR-positive MBCs were more likely to recur compared with ER/PR-negative tumors that showed a lower recurrence rate (43.5% vs. 25.5%, respectively). Our study could not find a statistical significance between ER/PR status and patient age, nodal status, follow-up duration, tumor differentiation, and survival status.

Table 3 shows that the different combinations of ER/PR receptor expression in the MBCs in our population. The majority of cases were negative for both ER and PR (67.1%).

Table 4 shows that the association of different combinations of ER and ER with clinicopathological parameters. Cases that were negative for both ER and PR were associated with a higher age, grade, and T- and N-stage. Conversely, cases that were ER positive and PR negative were significantly associated with higher recurrence and shorter survival.

Table 5 shows that the association between clinicopathological parameters and HER2/neu expression. Patients with positive HER2/neu expression had a higher age, whereas those with negative HER2/neu expression had higher tumor grade and N-stage. Conversely, HER2/neu-positive tumors had higher recurrence rates.

**Figure 3:**
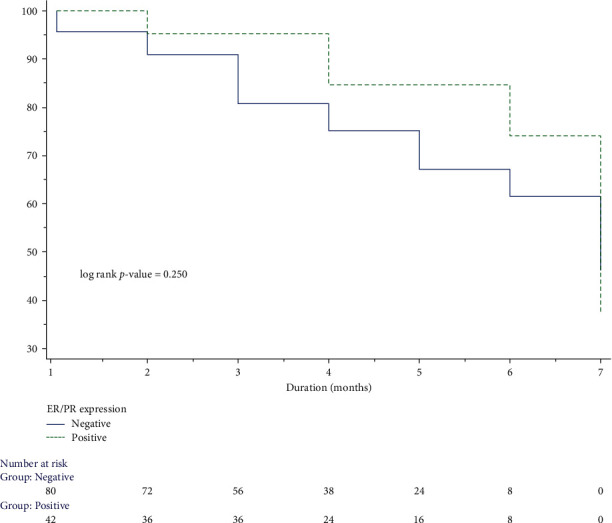
Association of estrogen/progesterone receptor expression with survival status by Kaplan–Meier analysis.

Figure 3 reveals the association of ER/PR expression with survival using the Kaplan–Meier curve. No significant association was found between the survival of ER/PR-positive and ER/PR-negative breast cancer patients.

## 4. Discussion

This study was conducted to determine the significance of HR status (ER/PR) in patients with MBC. Although MBCs are typically triple negative, approximately 32.9% of MBC cases in our population expressed positive ER/PR HR status. Moreover, our study demonstrated a statistically significant correlation between ER/PR expression and a few clinicopathological features such as tumor laterality, tumor size, tumor grade, tumor stage, and recurrence. We concluded that positive ER/PR MBCs were more likely to be observed on the left side of the breast and were smaller in size than ER/PR-negative MBCs. Furthermore, ER/PR-positive MBCs were more likely to be of lower grade than ER/PR-negative MBCs (grade 3 56.5% vs 85.1%, respectively). Similarly, ER/PR-positive MBCs presented at a lower stage than ER/PR-negative MBCs (T1- 16.7% vs 11.8%, T2- 66.7% vs 26.5%, T3- 16.7% vs 61.8%, respectively). In addition, ER/PR-positive MBCs showed a higher recurrence rate than ER/PR-negative MBCs (43.5% vs 25.5%, respectively). Therefore, we concluded that ER/PR-positive MBCs were probably of smaller size, lower grade, stage, and higher recurrence rate than ER/PR-negative MBCs, but no statistically significant survival difference was found between the two subgroups.

The mean age of patients with MBC in our population was found to be 52.18 ± 12.19 years, which was consistent with previous literature [11]. We also concluded that these tumors tended to be of higher grade and stage with low nodal involvement. These findings corroborate with previous studies stating that MBCs usually present at a higher stage and grade owing to its worst prognosis [11].

IHC is an integral tool for the diagnosis of MBC and determination of the management course with regard to HR status. Although the majority (>90%) of MBCs are triple negative, there is an atypical subtype that shows positivity for HR [12]. HR positivity in nonmetaplastic breast cancers is considered to be a good prognostic indicator and is associated with better outcome; however, some studies have proposed that positive HR status may not be associated with better prognosis in MBC [13]. Lim et al. [14] conducted a study and supported the idea that the nontriple-negative MBC (NTNMBC) subgroup was associated with a worse prognosis than triple-negative MBC (TNMBC). Moreover, they concluded that NTNMBCs showed rapid disease progression after relapse compared with TNMBCs, indicating that triple negative hormone status is a better prognostic indicator in MBC [14]. A few other studies conducted before on the significance of HR status concluded that positive or negative HR status has no significant difference and does not affect the overall prognosis of the disease [15]. Another study previously corroborated that HR status was not associated with the overall prognosis of MBC [16]. However, other studies support the idea that TNMBC has a worse prognosis than HR-positive MBC [17]. A study conducted by He et al. [18] on the prognosis of MBCs of different subtypes and found that TNMBCs were associated with a worse prognosis than TNBC, whereas NTNMBCs showed a similar prognosis as TNBC. In our study, we found that ER/PR-positive MBCs had better overall survival (73.9%) than ER/PR-negative MBCs (70.2%), but this difference was not statistically significant. Hence, further studies are required to understand the prognostic significance of HR status in MBC. Hu et al. [19] conducted a study on MBC and compared the clinicopathological characteristics and prognosis among different molecular subtypes. They concluded that the molecular subtype did not correlate with better survival.

In a study conducted previously by Abada et al. [20] on the prognostic relevance of ER receptor status in MBC, they found that the ER receptor positive expression rate in their study was much lower (12%). Moreover, although ER-positive tumors were more likely to have a smaller tumor size of 2.5 cm than ER-negative MBCs, these findings were not statistically significant. Furthermore, no statistically significant difference was found between ER-positive and ER-negative tumors regarding patient age, tumor stage, tumor grade, nodal involvement, or histological subtype.

Although in our study, no statistically significant association was observed between HR status and the morphological subtype of MBC, Rakha et al. [21] proposed that positive HR is most commonly positive in squamous carcinoma. A study conducted in India by Damera et al. [12] on MBC found that the ratio of ER/PR-positive MBC in their study was 39.3%, which was close to our finding of 32.9%. Moreover, their study demonstrated an association between TNMBCs and sarcomatoid histology (66.7%). Furthermore, they concluded that NTNMBCs displayed a more progressive disease course than TNMBCs and that NTNMBCs showed a lower percentage of disease-free survival (46.2%) than TNMBCs (66.7%), suggesting that the NTN hormone status is a worse prognostic indicator than the triple-negative hormone status. In our study, HER2/neu negativity in MBC was associated with higher grade and N-stage. The prognostic significance of HR status in MBC is still controversial due to varying findings; however, it is evident from the above findings that determination of HR status is integral in MBC as it may help in identifying the course of treatment.

MBC is a rare aggressive tumor that accounts for only 0.2%–5% of all breast cancers [22]. From 1973 to 2015, less than 10,000 cases of MBC were reported in the USA [23]. In Pakistan, exact data are not available owing to the lack of cancer registries; however, a hospital-based incidence rate of 1.92% has been reported [24].

Apart from histological parameters, the molecular profile of Asian breast cancer differs from that of the western/Caucasian population. Breast cancer in the Asian population had a higher prevalence of HER2/neu expression and TP53 mutations in ER-positive tumors. Moreover, immune scores were also higher, underscoring the relevance of immune checkpoint inhibitors among treatment options [25]. Another comparative study revealed that the Asian women had a higher incidence of young-age breast cancer, typically ER-negative than the white women [26]. A study conducted in Indian women compared the risk factors for developing breast cancer in ER-positive and ER-negative tumors. They found that a higher body mass index (BMI) was associated with ER-negative tumors in premenopausal women, whereas late marriage was associated with both ER-positive and ER-negative tumors. Similarly, breast feeding and physical activity were protective against both ER-positive and ER-negative tumors [27]. Similar to Asians, low HR positivity was observed in Chinese women with breast cancer. In a study involving 1,052 Chinese breast cancers, ER positivity was observed in 53% and 61.6% pre- and postmenopausal women with breast cancer [28].

A comprehensive review of the molecular features of MBCs revealed that they express more epithelial-to-mesenchymal-transition (EMT) markers, epidermal growth factor (EGFR) overexpression (34%), phosphoinositide 3-kinase (PIK3CA) mutations (47%), aberrant beta-catenin expression (92%), programmed death ligand 1 (PDL1) expression (46%), and TP53 mutations (64%) [29].

## 5. Conclusion

MBC is a rare and unique breast neoplasm. These tumors are usually of larger size, higher grade, and stage with a paucity of nodal involvement. MBC was found to be triple negative in most cases, but a significant proportion were HR (ER/PR) positive. The HR positivity rate in our study population was 32.9%. Moreover, we found an association between HR status and various clinicopathological features, indicating that HR status is a significant predictor of MBC prognosis. In addition, we found a statistically significant association between HR status and tumor size, grade, stage, and recurrence rate. This suggests that HR status is an important prognostic tool in the management of MBC.

## Figures and Tables

**Table 1:** Clinicopathological parameters of population under study.

Clinicopathological parameters	Values
Age (years); Mean ± SD	52.18 ± 12.19
Age groups	
≤35 years, *n* (%)	16 (11.4)
36–50 years, *n* (%)	52 (37.1)
>50 years, *n* (%)	72 (51.4)
Follow up duration (months); Mean ± SD	4.14 ± 1.88
Tumor laterality	
Right breast, *n* (%)	78 (55.7)
Left breast, *n* (%)	62 (44.3)
Type of biopsy	
Trucut, *n* (%)	48 (34.3)
Modified radical mastectomy, *n* (%)	40 (28.6)
Breast conversion surgery, *n* (%)	22 (15.7)
Simple mastectomy, *n* (%)	30 (21.4)
Tumor size (cm), *n* = 92; Mean ± SD	5.99 ± 3.44
Tumor (T) stage, *n* = 92	
T1, *n* (%)	12 (13)
T2, *n* (%)	34 (37)
T3, *n* (%)	46 (50)
Tumor grade	
Grade 2, *n* (%)	34 (24.3)
Grade 3, *n* (%)	106 (75.7)
Squamous differentiation	
Present, *n* (%)	96 (68.6)
Absent, *n* (%)	44 (31.4)
Chondroid differentiation	
Present, *n* (%)	8 (5.7)
Absent, *n* (%)	132 (94.3)
Spindle cell differentiation	
Present, *n* (%)	16 (11.4)
Absent, *n* (%)	124 (88.6)
Nodal (N) stage, *n* = 92	
N0, *n* (%)	52 (56.5)
N1, *n* (%)	18 (19.6)
N2, *n* (%)	9 (9.8)
N3, *n* (%)	13 (14.1)
Recurrence	
Yes, *n* (%)	44 (31.4)
No, *n* (%)	96 (68.6)
Survival status	
Alive, *n* (%)	100 (71.4)
Expired, *n* (%)	40 (28.6)
ER/PR	
Positive, *n* (%)	46 (32.9)
HER2/neu	
Positive, *n* (%)	32 (22.9)
Negative, *n* (%)	108 (77.1)

SD: standard deviation; T: tumor; N: nodal; ER: estrogen receptor; PR: progesterone receptor.

**Table 2:** Association of clinicopathological parameters with estrogen and progesterone receptor expression.

Clinicopathological parameters	Values		*p*-Value
	ER/PR expression		
	Positive	Negative	
Age (years), Mean ± SD ^*∗*^	53.17 ± 13.16	51.70 ± 11.73	0.504
Age group ^*∗∗*^			
≤35 years, *n* (%)	2 (4.3)	14 (14.9)	0.181
36–50 years, *n* (%)	18 (39.1)	34 (36.2)	
>50 years, *n* (%)	26 (56.5)	46 (48.9)	
Follow-up duration (months), Mean ± SD ^*∗*^	4.52 ± 1.88	3.95 ± 1.86	0.096
Tumor laterality ^*∗∗*^			
Right breast, *n* (%)	20 (43.5)	58 (61.7)	0.041 ^*∗∗∗*^
Left breast, *n* (%)	26 (56.5)	36 (38.3)	
Tumor size (cm), *n* = 92 ^*∗*^	4.20 ± 1.88	6.62 ± 3.43	0.003 ^*∗∗∗*^
Tumor (T) stage, *n* = 92 ^*∗∗*^			
T1, *n* (%)	4 (16.7)	8 (11.8)	<0.001 ^*∗∗∗*^
T2, *n* (%)	16 (66.7)	18 (26.5)	
T3, *n* (%)	4 (16.7)	42 (61.8)	
Tumor grade ^*∗∗*^			
Grade 2, *n* (%)	20 (43.5)	14 (14.9)	<0.001 ^*∗∗∗*^
Grade 3, *n* (%)	26 (56.5)	80 (85.1)	
Squamous differentiation ^*∗∗*^			
Present, *n* (%)	30 (65.2)	66 (70.2)	0.550
Absent, *n* (%)	16 (34.8)	28 (29.8)	
Chondroid differentiation ^*∗∗*^			
Present, *n* (%)	4 (8.7)	4 (4.3)	0.439
Absent, *n* (%)	42 (91.3)	90 (95.7)	
Spindle cell differentiation ^*∗∗*^			
Present, *n* (%)	4 (8.7)	12 (12.8)	0.477
Absent, *n* (%)	42 (91.3)	82 (87.2)	
Nodal (N) stage, *n* = 92, *n* (%) ^*∗∗*^			
N0, *n* (%)	14 (58.3)	38 (55.9)	0.107
N1, *n* (%)	4 (16.7)	14 (20.8)	
N2, *n* (%)	5 (20.8)	4 (5.9)	
N3, *n* (%)	1 (4.2)	12 (17.6)	
Recurrence, *n* (%) ^*∗∗*^			
Yes, *n* (%)	20 (43.5)	24 (25.5)	0.032 ^*∗∗∗*^
No, *n* (%)	26 (56.5)	70 (74.5)	
Survival status, *n* (%) ^*∗∗*^			
Alive, *n* (%)	34 (73.9)	66 (70.2)	0.649
Expired, *n* (%)	12 (26.1)	28 (29.8)	

^*∗*^Independent *t*-test was applied,  ^*∗∗*^*Chi*-square/Fisher's exact test was applied,  ^*∗∗∗*^*p*-value significant as <0.05. ER: estrogen receptor; PR: progesterone receptor; SD: standard deviation; T: tumor; N: nodal.

**Table 3:** Different combinations of estrogen/progesterone expression in metaplastic carcinoma.

ER/PR expression	Values
ER positive, PR positive, *n* (%)	24 (17.1)
ER positive, PR negative, *n* (%)	18 (12.9)
ER negative, PR positive, *n* (%)	4 (2.9)
ER negative, PR negative, *n* (%)	94 (67.1)

ER: estrogen receptor; PR: progesterone receptor.

**Table 4:** Association of clinicopathological parameters with different combinations of estrogen and progesterone receptor expression.

Clinicopathological parameters	Values				*p*-Value
	ER/PR expression				
	ER+, PR+	ER+, PR−	ER−, PR+	ER−, PR−	
Age (years), Mean ± SD ^*∗*^	52.75 ± 10.60	56.00 ± 16.54	43.00 ± 0.00	51.70 ± 11.73	0.239
Age group ^*∗∗*^					
≤35 years, *n* (%)	0 (0)	2 (11.1)	0 (0)	14 (14.9)	0.045 ^*∗∗∗*^
36–50 years, *n* (%)	10 (41.7)	4 (22.2)	4 (100)	34 (36.2)	
>50 years, *n* (%)	14 (58.3)	12 (66.7)	0 (0)	46 (48.9)	
Follow-up duration (months), Mean ± SD ^*∗*^	4.41 ± 2.06	4.44 ± 1.68	5.50 ± 1.73	3.95 ± 1.86	0.268
Tumor laterality ^*∗∗*^					
Right breast, *n* (%)	12 (50)	8 (44.4)	0 (0)	58 (61.7)	0.050 ^*∗∗∗*^
Left breast, *n* (%)	12 (50)	10 (55.6)	4 (100)	36 (38.3)	
Tumor size (cm), *n* = 92 ^*∗*^	3.00 ± 1.26	7.83 ± 3.35	NA	6.62 ± 3.43	<0.001 ^*∗∗∗*^
Tumor (T) stage, *n* = 92 ^*∗∗*^					
T1, *n* (%)	4 (22.2)	0 (0)	0 (0)	8 (11.8)	<0.001 ^*∗∗∗*^
T2, *n* (%)	14 (77.8)	2 (33.3)	0 (0)	18 (26.5)	
T3, *n* (%)	0 (0)	4 (66.7)	0 (0)	42 (61.8)	
Tumor grade ^*∗∗*^					
Grade 2, *n* (%)	8 (33.3)	8 (44.4)	4 (100)	14 (14.9)	<0.001 ^*∗∗∗*^
Grade 3, *n* (%)	16 (66.7)	10 (55.6)	0 (0)	80 (85.1)	
Squamous differentiation ^*∗∗*^					
Present, *n* (%)	14 (58.3)	12 (66.7)	4 (100)	66 (70.2)	0.426
Absent, *n* (%)	10 (41.7)	6 (33.3)	0 (0)	28 (29.8)	
Chondroid differentiation ^*∗∗*^					
Present, *n* (%)	4 (16.7)	0 (0)	0 (0)	4 (4.3)	0.100
Absent, *n* (%)	20 (83.3)	18 (100)	4 (100)	90 (95.7)	
Spindle cell differentiation ^*∗∗*^					
Present, *n* (%)	0 (0)	4 (22.2)	0 (0)	12 (12.8)	0.102
Absent, *n* (%)	24 (100)	14 (77.8)	4 (100)	82 (87.2)	
Nodal (N) stage, *n* = 92, *n* (%) ^*∗∗*^					
N0, *n* (%)	14 (77.8)	0 (0)	0 (0)	38 (55.9)	<0.001 ^*∗∗∗*^
N1, *n* (%)	4 (22.2)	0 (0)	0 (0)	14 (20.6)	
N2, *n* (%)	0 (0)	6 (100)	0 (0)	4 (5.9)	
N3, *n* (%)	0 (0)	0 (0)	0 (0)	12 (17.6)	
Recurrence, *n* (%) ^*∗∗*^					
Yes, *n* (%)	4 (16.7)	12 (66.7)	4 (100)	24 (25.5)	<0.0001 ^*∗∗∗*^
No, *n* (%)	20 (83.3)	6 (33.3)	0 (0)	70 (74.5)	
Survival status, *n* (%) ^*∗∗*^					
Alive, *n* (%)	24 (100)	10 (55.6)	0 (0)	66 (70.2)	<0.001 ^*∗∗∗*^
Expired, *n* (%)	0 (0)	8 (44.4)	4 (100)	28 (29.8)	

^*∗*^One-way ANOVA was applied,  ^*∗∗*^*Chi*-square/Fisher's exact test was applied,  ^*∗∗∗*^*p*-value significant as <0.05. ER: estrogen receptor; PR: progesterone receptor; SD: standard deviation; T: tumor; N: nodal.

**Table 5:** Association of clinicopathological parameters with HER2/neu expression.

Clinicopathological parameters	Values		*p*-Value
	HER2/neu expression		
	Positive	Negative	
Age (years), Mean ± SD ^*∗*^	56.12 ± 12.11	51.01 ± 12.03	0.037 ^*∗∗∗*^
Age group ^*∗∗*^			
≤35 years, *n* (%)	0 (0)	16 (14.8)	0.042 ^*∗∗∗*^
36–50 years, *n* (%)	12 (37.5)	40 (37)	
>50 years, *n* (%)	20 (62.5)	52 (48.1)	
Follow-up duration (months), Mean ± SD ^*∗*^	4.31 ± 1.99	4.09 ± 1.85	0.564
Tumor laterality ^*∗∗*^			
Right breast, *n* (%)	6 (18.8)	72 (66.7)	<0.001 ^*∗∗∗*^
Left breast, *n* (%)	26 (81.3)	36 (33.3)	
Tumor size (cm), *n* = 92 ^*∗*^	5.33 ± 3.44	6.09 ± 3.45	0.480
Tumor (T) stage, *n* = 92 ^*∗∗*^			
T1, *n* (%)	0 (0)	12 (15)	0.073
T2, *n* (%)	8 (66.7)	26 (32.5)	
T3, *n* (%)	4 (33.3)	42 (52.5)	
Tumor grade ^*∗∗*^			
Grade 2, *n* (%)	12 (37.5)	22 (20.4)	0.047 ^*∗∗∗*^
Grade 3, *n* (%)	20 (62.5)	86 (79.6)	
Squamous differentiation ^*∗∗*^			
Present, *n* (%)	32 (100)	64 (59.3)	<0.001 ^*∗∗∗*^
Absent, *n* (%)	0 (0)	44 (40.7)	
Chondroid differentiation ^*∗∗*^			
Present, *n* (%)	0 (0)	8 (7.4)	0.198
Absent, *n* (%)	32 (100)	100 (92.6)	
Spindle cell differentiation ^*∗∗*^			
Present, *n* (%)	0 (0)	16 (14.8)	0.023 ^*∗∗∗*^
Absent, *n* (%)	32 (100)	92 (85.2)	
Nodal (N) stage, *n* = 92, *n* (%) ^*∗∗*^			
N0, *n* (%)	4 (33.3)	48 (60)	0.013 ^*∗∗∗*^
N1, *n* (%)	4 (33.3)	14 (17.5)	
N2, *n* (%)	4 (33.3)	6 (7.5)	
N3, *n* (%)	0 (0)	12 (15)	
Recurrence, *n* (%) ^*∗∗*^			
Yes, *n* (%)	20 (62.5)	24 (22.2)	<0.001 ^*∗∗∗*^
No, *n* (%)	12 (37.5)	84 (77.8)	
Survival status, *n* (%) ^*∗∗*^			
Alive, *n* (%)	20 (62.5)	80 (74.1)	0.203
Expired, *n* (%)	12 (37.5)	28 (25.9)	

^*∗*^Independent *t*-test was applied,  ^*∗∗*^*Chi*-square/Fisher's exact test was applied,  ^*∗∗∗*^*p*-value significant as <0.05. ER: estrogen receptor; PR: progesterone receptor; SD: standard deviation; T: tumor; N: nodal.

## Data Availability

Data files are available on request. Please contact author, Atif Ali Hashmi (atifhashmi345@gmail.com) for data requests.

## Additional Points

*Limitations*. Our study had a few limitations. First, our study was a single-centered study and had a limited sample size. Moreover, the risk factors for the disease were not evaluated, and a more comprehensive molecular study was lacking. The response to treatment was also not evaluated. A prospective study on patients with MBC is a challenge due to its rarity and aggressive nature; however, it is important to conduct a multicentered study to better understand the disease, its subtypes, and its molecular alternation, which may help in designing a more effective treatment of the disease and improve its prognosis. Moreover, molecular signaling pathways such as nitric oxide synthase (NOS) signaling, Wnt/*β*-catenin signaling, altered immune response, PI3K signaling, and cell cycle dysregulation were not evaluated in our study, which could have provided more information about the role of HR status in MBC. Similarly, besides histological organization, cellular origins of MBC, molecular alterations, and the role of radiation therapy can also aid in understanding HR status.
